# Fine particulate matter 2.5 exerted its toxicological effect by regulating a new layer, long non-coding RNA

**DOI:** 10.1038/s41598-017-09818-6

**Published:** 2017-08-24

**Authors:** Qiansheng Huang, Yulang Chi, Junjun Deng, Yiyao Liu, Yanyang Lu, Jinsheng Chen, Sijun Dong

**Affiliations:** 1 Center for Excellence in Regional Atmospheric Environment, Institute of Urban Environment, Chinese Academy of Sciences, Xiamen, 361021 China; 2 Key Laboratory of Urban Environment and Health, Institute of Urban Environment, Chinese Academy of Sciences, Xiamen, 361021 China

## Abstract

Fine particulate matter (PM2.5) exposure, especially to its organic components, induces adverse health effects on the respiratory system. However, the molecular mechanisms have still not been fully elucidated. Long non-coding RNA (lncRNA) is involved in various physio-pathological processes. In this study, the roles of lncRNA were investigated to reveal the toxicology of PM2.5. Organic extracts of PM2.5 from Nanjing and Shanghai cities were adopted to treat human bronchial epithelial cell lines (BEAS-2B and A549). RNA sequencing showed that the lncRNA functioned as antisense RNA, intergenic RNA and pre-miRNA. The mRNA profiles were also altered after exposure. PM2.5 from Nanjing showed a more serious impact than that from Shanghai. In detail, higher expression of n405968 was positively related to the elevated mRNA levels of inflammatory factors (IL-6 and IL-8). Increasing levels of metastasis associated lung adenocarcinoma transcript 1 (MALAT1) were positively associated with the induced epithelial-mesenchymal transition (EMT) process. Similar response was observed between both cell lines. The higher content of polycyclic aromatic hydrocarbons (PAHs) is likely to contribute to higher toxicity of PM2.5 from Nanjing than that from Shanghai. Antagonism of aryl hydrocarbon receptor (AHR) or inhibition of CYP1A1 diminished the effects stimulated by PM2.5. Our results indicated that lncRNAs could be involved in the toxicology of PM2.5 through regulating the inflammation and EMT process.

## Introduction

Serious air pollution, especially fine particulate matter (PM2.5, particles with an aerodynamic diameter of 2.5 μm or less), is a major health concern^1–3^. PM2.5 has adverse impacts on the respiratory system. Epidemiological studies have shown that PM2.5 is associated with abnormal lung function^4^, lung infection^5^, and lung cancer^6^. Long-term improvement of air quality was associated with better lung-function growth in children^7^. By contrast, toxicological data have been largely insufficient for understanding the molecular mechanisms. PM2.5 is composed of organic carbon (OC), elemental carbon (EC), water-soluble inorganic ions and metal elements. OC consists of various organic compounds, including PAHs, PCBs, phthalate esters, aldehydes, ketones, benzene and so on. Crustal elements (K, Fe, Mg, Al, Ca) and toxic heavy metals (Hg, Cr, Cu, Pb, Zn) are both present in PM2.5. water-soluble inorganic ions contains NO_3_^−^, SO_4_^2−^, NH_4_^+^, Cl^−^. The constituents of PM2.5 are regional and time specific^8–10^. The components of PM2.5 largely affected its toxic outcome^11^. The particle contributed to the toxicityin a way which is similar to that of nanomaterials^12, 13^. In addition, organic chemicals adsorbed on the surface possess appreciable toxicity^14, 15^.

Long noncoding RNA (lncRNA), which has more than 200 nucleotides in length, is commonly involved in physiological and pathological process^16^. Growing numbers of lncRNAs are being continuously discovered, and they play important roles in cancers and other diseases^17–19^. The expression levels of MALAT1 (metastasis associated lung adenocarcinoma transcript 1) and AFAP1-AS1 (actin filament associated protein 1 antisense RNA 1) were both significantly up-regulated, whereas TUG1 (taurine-upregulated gene 1) and HMlincRNA 717 were both down-regulated in non-small cell lung cancer (NSCLC)^20^. The usefulness of lncRNA as a non-invasive marker is expected in lung disease^21^.

LncRNA functions in response to environmental stimuli. MALAT1 and HOTAIR were both involved in the epithelial-mesenchymal transition (EMT) induced by cigarette smoke extract^22, 23^. Bisphenol-A and diethylstilbestrol could alter the expression of HOTAIR *in vitro* and *in vivo* ^24^. LncRNA could be a potential indicator of the toxicity induced by chemical exposure^25^.

To the best of our knowledge, lncRNA has not yet been considered in toxicological studies of PM2.5. In this study, organic extracts of PM2.5 from Nanjing and Shanghai cities were both adopted to treat both BEAS-2B and A549 cells. These two cell lines have been widely used in toxicological studies of pollutants on the respiratory system^26–28^. Differentially expressed lncRNAs were co-analyzed with their corresponding mRNAs. The expressions and phenotypes of several lncRNA-mRNA pairs related to the pathology of lung cancer were further studied upon exposure. The mediating role of the AHR signaling pathway was shown in the study and was also evidenced by the presence of 16 priority polycyclic aromatic hydrocarbons (PAHs) in PM2.5.

## Results

### Differentially expressed lncRNAs after exposure to PM2.5

**Figure 1:**
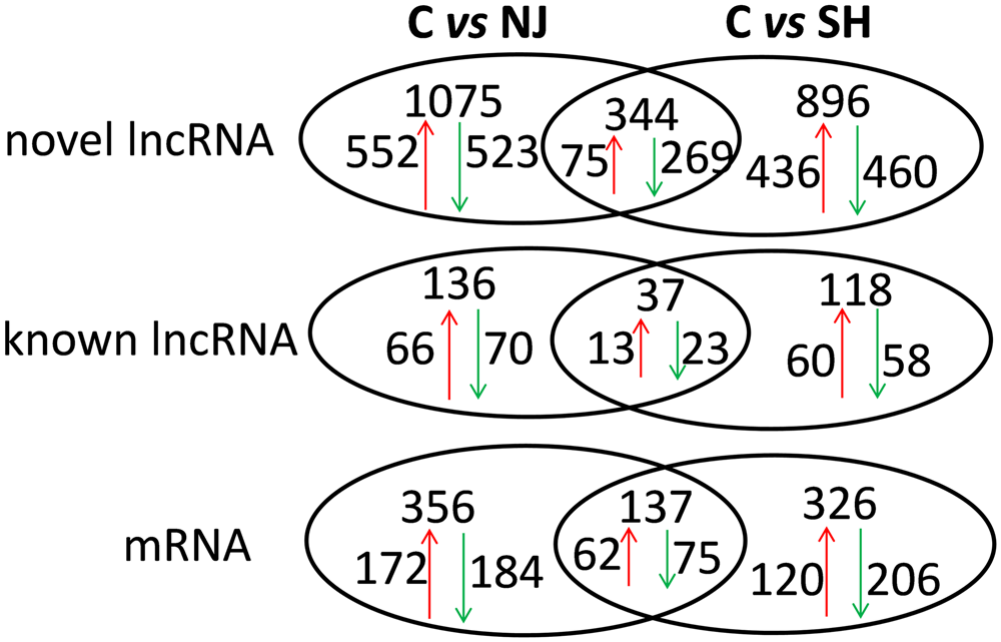
Comparison of the differentially expressed RNAs. C: control group, NJ: organic extract of PM2.5 from Nanjing city, SH: organic extract of PM2.5 from Shanghai city.

To ensure that transcriptional changes induced by exposure were related to RNA expression rather than cell viability, the effect of PM2.5 on cell viability was first determined by MTT assay (supplementary information 1, Figure S1). The results showed that the dose used in our study (5 μg/mL) did not have a significant influence on the cell viability of BEAS-2B or A549 cells. RNA sequencing was conducted to detect the profiles of RNA transcripts from BEAS-2B cells after exposure at 5 μg/mL. A total of 6,624, 6,977 and 6,055 noncoding transcripts were obtained from control, NJ-exposed and SH-exposed BEAS-2B cells, respectively. We then distinguished the differentially expressed lncRNAs between groups (Fig. 1). In total, the expression levels of 618 lncRNAs were up-regulated, and 593 lncRNAs were down-regulated after exposure to Nanjing PM2.5, compared to controls. The numbers were 496 and 518 for the SH-exposed group, which was smaller than the NJ group. These two groups shared 381 common lncRNAs with the same expression patterns. Most of the differentially expressed lncRNAs were novel, and the known lncRNAs numbered relatively few. In contrast, A total of 356 and 326 coding genes were altered at the mRNA level after exposure to PM2.5 from NJ and SH, respectively. The mRNA transcripts of 137 genes were altered after exposure to PM2.5 from both cities. Detailed information on individual lncRNAs and mRNAs are available in supplementary information 2.

### Functional prediction of differentially expressed lncRNAs

The predicted functions of differentially expressed genes were further categorized (Table 1). A total of 1,633 lncRNAs were predicted to be the antisense of coding transcripts. In contrast, the numbers were 253 and 4,340 for pre-miRNA and intergenic lncRNA, respectively. Most of the differentially expressed lncRNAs were predicted to be intergenic genes. They are located upstream or downstream of the coding genes. The numbers of antisense lncRNAs were 33 and 23 in the NJ and SH PM2.5-treated groups, respectively. They could form the antisense lncRNA-mRNA duplex. Novel pre-miRNA was also discovered from these differentially expressed lncRNAs.

**Table 1:** Classification of differentially expressed lncRNA.

	C *vs* NJ	C *vs* SH	common
Antisense	33	23	12
Up/downstream	377	320	117
PremiRNA_known	5	7	2
PremiRNA_novel	43	33	12
Family	1	1	1

Antisense: antisense to related coding genes; Up/down stream: upstream lncRNA, overlaps with the promoter regions or other cis-regulatory elements of their co-expressed coding genes; downstream lncRNA, the 3’UTRs or downstream regions of protein-coding genes.

### Functional analysis of differentially expressed mRNAs

The mRNA profiles were also studied after exposure. GO analysis showed that differentially expressed genes possessed various functions (supplementary information 1 Figure S2). The significantly enriched KEGG pathways are listed in supplementary information 1 Table S2. Both treatments shared the majority of pathways. They were mainly related to human disease, environmental information processing, and metabolism.

### LncRNA-mRNA pairs and their phenotypes

**Figure 2:**
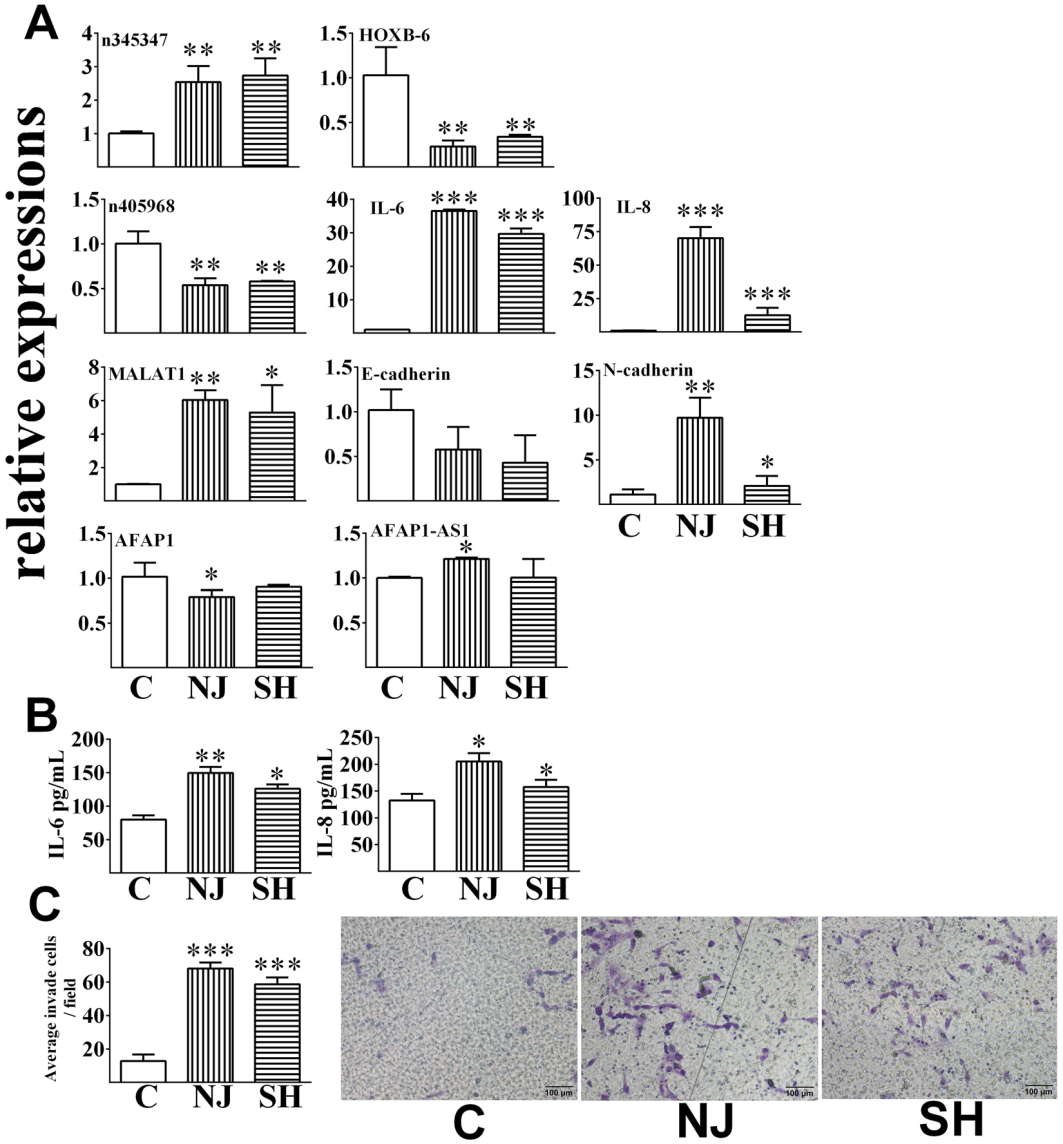
The relative expression levels of lncRNA-mRNA pairs and the related phenotypes of BEAS-2B cells upon exposure. (**A**) Relative expression levels after exposure for 48 h by real-time RT-PCR; (**B**) The levels of IL-6 and IL-8 in the cell culture media after exposure for 48 h, measured by ELISA; (**C**) The number of invaded cells after exposure measured using a transwell assay. All data are presented as the means ± SDs (n = 3). Independent-samples t-tests were used for data analysis, *p < 0.05, **p < 0.01, ***p < 0.001.

**Figure 3:**
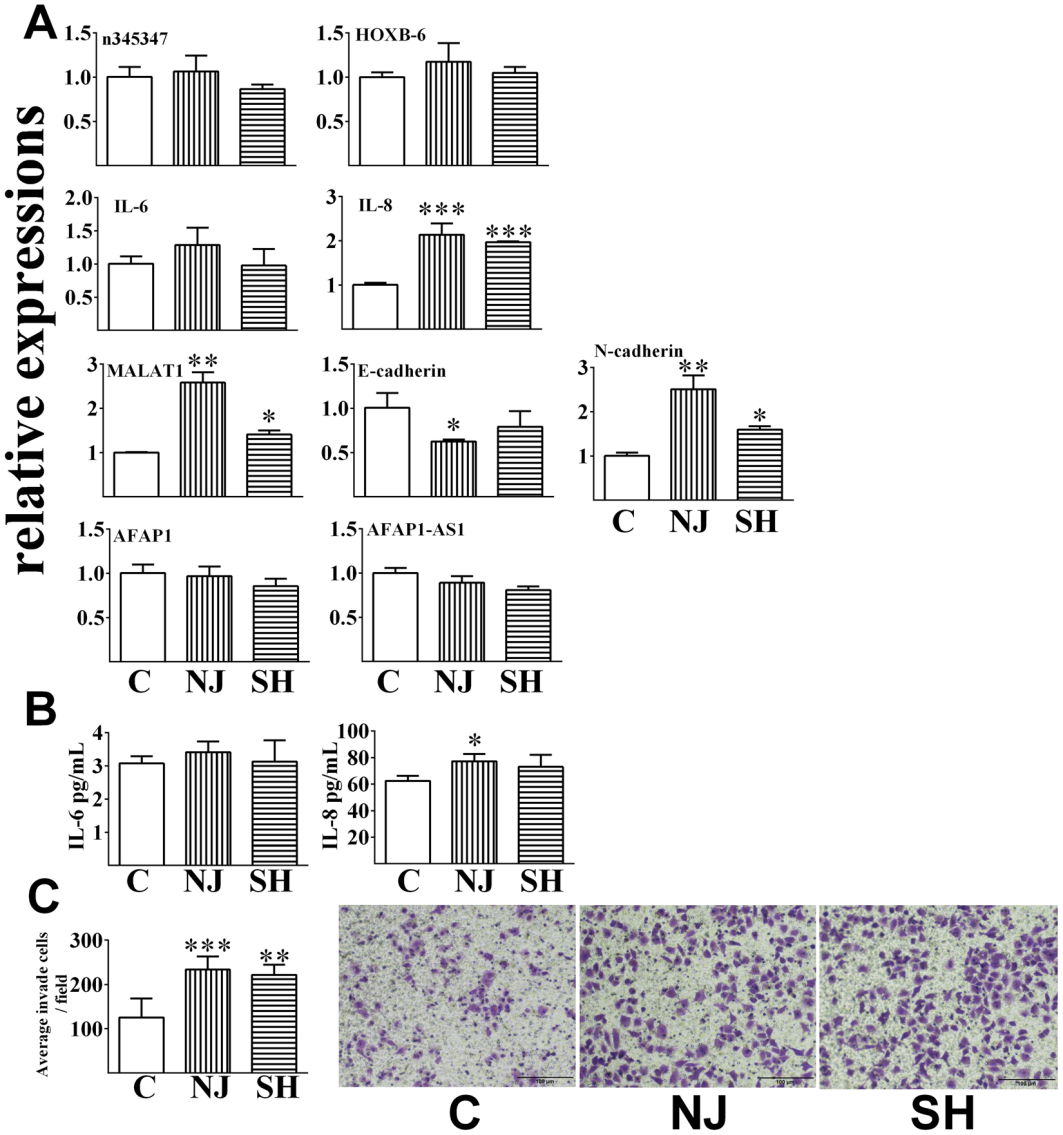
The relative expression levels of lncRNA-mRNA pairs and the related phenotypes of A549 cells upon exposure. (**A**) Relative expression levels after exposure for 48 h by real-time RT-PCR; (**B**) The levels of IL-6 and IL-8 in the cell culture media after exposure for 48 h, measured by ELISA; (**C**) The number of invaded cells after exposure measured using a transwell assay. All data are presented as the means ± SDs (n = 3). Independent-samples t-tests were used for data analysis, *p < 0.05, **p < 0.01, ***p < 0.001.

The levels of several lncRNA and mRNA pairs were further confirmed by RT-PCR (Fig. 2A). In BEAS-2B cells, the expression level of n345347 was significantly up-regulated upon exposure to PM2.5 from both cities. Its antisense gene (homeobox B6, HOXB-6) showed a reverse response to exposure, which decreased significantly upon exposure. The expression of n405968 was significantly down-regulated following either exposure. Abnormal expression of n405968 was related to the inflammatory response. The mRNA levels of interleukins (IL-6 and IL-8) were both significantly up-regulated upon exposure to PM2.5 from either city. Secreted IL-6 and IL-8 were also elevated in the cell culture media (Fig. 2B). The transcript of MALAT1 was markedly induced following either exposure. This increasing trend was positively related to the EMT process. Decreased levels of E-cadherin and elevated levels of N-cadherin were observed after exposure. The cell invasion ability was enhanced upon exposure (Fig. 2C). The expression level of AFAP1-AS1 increased, whereas the level of its sense gene AFAP1 decreased, following treatment with PM2.5 from Nanjing. In contrast, PM2.5 from Shanghai did not significantly affect the expression levels of this gene pair. The expression levels of related genes were also detected in A549 cells upon exposure (Fig. 3). Neither of the levels of n345347 and HOXB-6 was significantly influenced after exposure to PM2.5 from either city (Fig. 3A). The level of n405968 was not detectable in A549 cells. PM2.5 exposure did not significantly affect the level of IL-6. By contrast, the level of IL-8 significantly increased at both mRNA and protein levels which was similar to that occurred in BEAS-2B cells (Fig. 3B). Responses of MALAT1, E-cadherin and N-cadherin in A549 cells were also similar between these two cell types. The invasive ability of A549 cells was enhanced compared to control group upon exposure (Fig. 3C).

### Determination of PAH levels

The levels of sixteen priority PAHs were determined, and their total concentrations are presented in Table 2. Total PAH levels of PM2.5 in Nanjing (21.687 ng/m^3^) were higher than those in Shanghai (15.695 ng/m^3^). Total BaP equivalent concentrations in Nanjing were 53% higher than those in Shanghai.

**Table 2:** PAH contents of PM2.5 from Nanjing and Shanghai.

ng/m^3^	NJ	SH
Total PAHs	21.687	15.695
Total BaP equivalence	3.623	2.614

Total PAHs were the sums of 16 priority PAHs. Total BaP equivalence was the sum of the equivalences of individual PAHs.

### Mediating roles of the AHR signaling pathway on the effects of PM2.5

**Figure 4:**
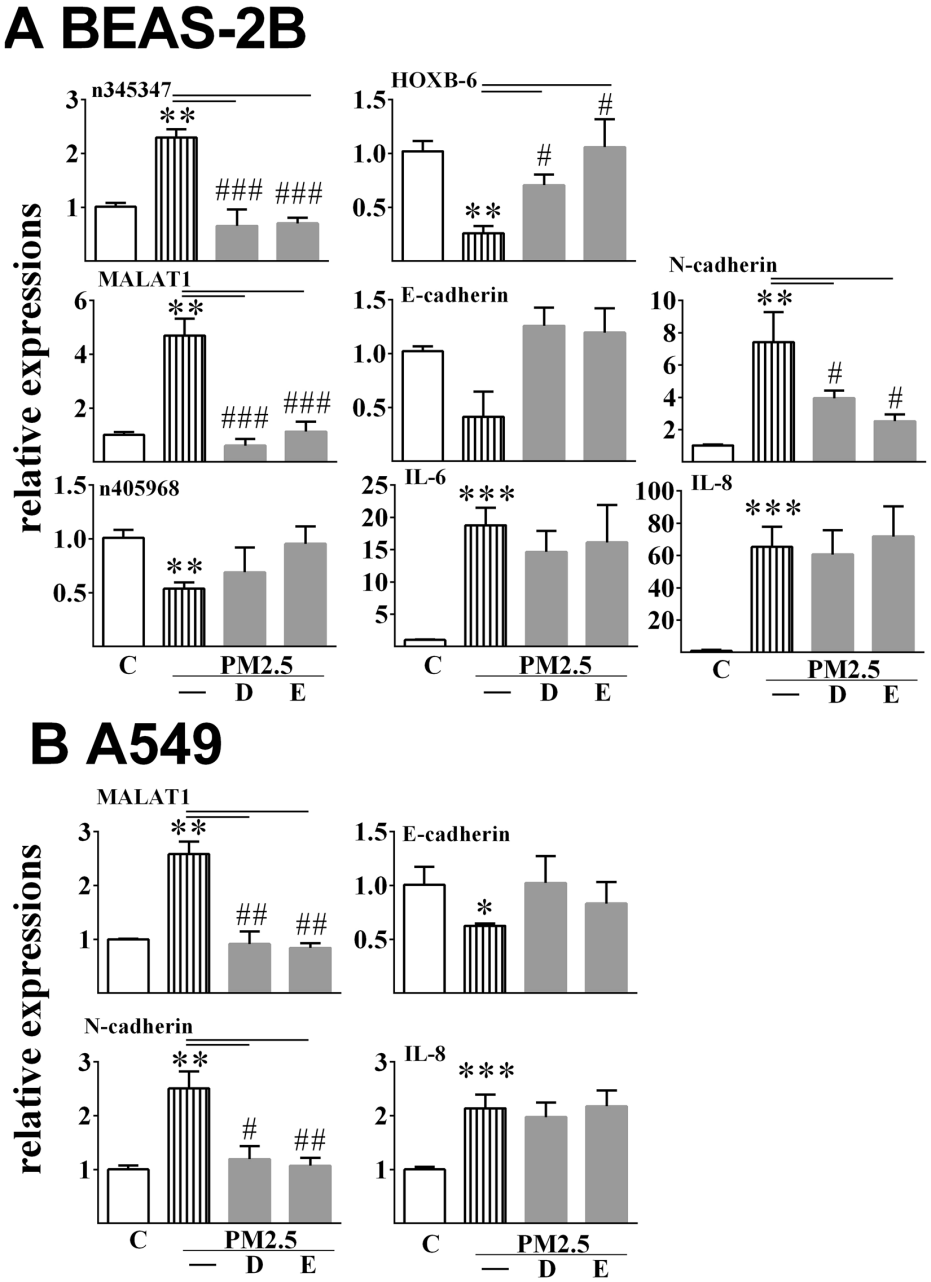
Effects of the antagonist on the relative expression levels of lncRNA and its related coding genes induced by PM2.5 in BEAS-2B cells (**A**) and A549 cells (**B**). All data are presented as the means ± SDs (n = 3). Independent-samples t-tests were used for data analysis, **p < 0.01 *vs* control group; ^#^ *p* < 0.05, ^##^ *p* < 0.01, ^###^ *p* < 0.001, PM2.5 single exposed group *vs* PM2.5/antagonist co-exposed group. DMF (**D**), antagonist to AHR; ellipticine (**E**), inhibitor of CYP1A1.

Cells were co-treated with Nanjing PM2.5 and AHR antagonist or CYP1A1 inhibitor (Fig. 4). Elevated levels of MALAT1 stimulated by PM2.5 significantly fell back to the level which was equal to that of control group after co-treatment in both cells. In accordance, the mRNA level of E-cadherin was higher in the co-exposed group than that in the single PM2.5-exposed group. The mRNA level of N-cadherin in the co-exposed group was significantly lower than in the single PM2.5-exposed group. Antagonism of AHR and inhibition of CYP1A1 both significantly diminished the stimulated effects of n345347 in BEAS-2B cells exposure to PM2.5. In the case of HOXB-6, both treatments led to the recovery of its expression in BEAS-2B cells. Neither of the chemicals affected the responses of n405968 and AFAP1-AS1 to PM2.5 exposure in both cells. Notably, the co-treatment did not significantly diminish the stimulatory effects of PM2.5 on the levels of both IL-6 and IL-8 in both cells. Similar trends were observed in co-treatment with PM2.5 from Shanghai.

## Discussion

The roles of lncRNAs in the toxicity of chemicals have been receiving attention recently. In this study, we detected the responses of lncRNAs to organic extracts of PM2.5 exposure in human bronchial epithelial cells. The lncRNA landscape in bronchial epithelial cells was obtained by RNA sequencing. LncRNA was widely involved in the toxicity of PM2.5. Previous studies of the toxicology of PM2.5 were all conducted at the level of DNA, mRNA, miRNA or protein. Our results provided a new layer of lncRNA to facilitate the understanding of PM2.5 toxicology. Differentially expressed lncRNAs were widely detected following exposure to PM2.5 from either city. Additionally, abnormal expression of lncRNA also occurred at the clinical level. Dysregulated lncRNA was widely observed in patients with lung diseases^29^.

In our study, two types of lung epithelial cells (BEAS-2B and A549) were adopted to determine the effects of organic components of PM2.5 on lung cells. PM2.5 has no significant toxicity to the viability of both cells at lower dose of 5 μg/mL. Inflammation and EMT are both involved in the etiology of lung cancer. LncRNA n405968 is the host gene of microRNA-155 (MIR155), which participates in inflammation^30^. In our study, the levels of IL-6 and IL-8 both increased in BEAS-2B cells after PM2.5 exposure. PM2.5 exposure also triggered the release of IL-8 in A549 cells. However, the level of IL-6 stayed at low levels after exposure. Regulation of n405968 might be one of the ways by which PM2.5 triggers the inflammatory response in BEAS-2B cells rather than in A549 cells. MALAT1 (also known as NEAT2) is involved in the regulation of cell cycle, cell migration, and cancer metastasis^22^. In our study, elevated levels of MALAT1 were positively associated with EMT processes, as indicated by the down-regulation of E-cadherin, up-regulation of N-cadherin, as well as the invasive ability of cells. PM2.5 likely triggers the EMT process by enhancing the expression of MALAT1. AFAP1-AS1 and its sense transcript AFAP1 showed reverse transcript patterns upon NJ PM2.5 exposure in BEAS-2B cells. LncRNA could function as an antisense transcript of the coding genes. In our study, AFAP1-AS1 and its antisense transcript AFAP1 showed reverse expression patterns upon PM2.5 exposure. A large amount of antisense lncRNA was predicted in our study. LncRNA (n345347) is the antisense to HOXB. HOXB acts as the transcription factor, and it is involved in lung development and related diseases, such as small cell lung cancers and bronchopulmonary sequestration^31, 32^.

Diverse sources of PM2.5 differ in their constituents and health outcomes^33, 34^. PM2.5 from Nanjing is predominantly from coal combustion whereas the major source is diesel and gasoline exhaust in Shanghai (as demonstrated by the local environmental protection bureaus: Nanjing: http://hbj.nanjing.gov.cn/; Shanghai: http://www.sepb.gov.cn). Concentrations of total PAHs and the corresponding benzopyrene (BaP) equivalences were higher in Nanjing than in Shanghai. Although the content in PM is very small, PAHs were regarded as the primary toxic constituents of PM and contributed to a higher risk of lung cancer^35–37^. In accordance, more differentially expressed lncRNAs were detected upon exposure to PM2.5 from Nanjing than from Shanghai. Levels of secreted inflammatory factors and their invasion ability were higher after treatment with PM2.5 from Nanjing than that from Shanghai. PAHs functioned through AHR receptor, which is an important mediator of the toxicity of organic chemicals. In this study, we found that this receptor-mediated effect also existed in the regulation of lncRNA expressions by PM2.5. Antagonism of AHR or inhibition of CYP1A1 obviously diminished the influence of PM2.5 on lncRNA-mRNA expression pairs. AHR mediated the carcinogenic effect stimulated by pollutants^38^.

In conclusion, our study provided new information on the toxic mechanism by which PM2.5 exerted its adverse health impacts through affecting the expressions of lncRNAs. LncRNA was involved in the inflammatory reaction and in the EMT process of lung epithelial cells. The AHR-CYP1A1 pathway mediated the effects of PAH-bound PM2.5 on lncRNA. This molecule could be a valuable target for revealing the toxicological mechanism of PM2.5.

## Materials and Methods

### Sample collection and extraction

PM2.5 was collected from Nanjing city (Gulou Campus, Nanjing University, 15 m height, 32° 055′ N, 118° 776′ E) and Shanghai city (Shanghai Academy of Environmental Sciences, 18 m height, 31°172′N, 121°425′ E) in the Yangtze River Delta region of China in Autumn 2014. The sampling period was on weekdays from November 6th to 17th. The climate parameters were as follows: average temperature (Nanjing, 21.3 ± 2.7 °C; Shanghai, 21.0 ± 2.9 °C) and barometric pressure (Nanjing, 765.1 ± 2.1 mm Hg; Shanghai, 765.4 ± 2.5 mm Hg). PM2.5 was captured on quartz filters (22.5 cm × 18.2 cm, Whatman Company, Kent, UK) at a flow rate of 1.05 m^3^/min by a large-size volume active sampler (HiVol 3000 air sampler, Ecotech, Australia). The average mass of PM2.5 in each membrane was 135.5 ± 37.6 mg and 121.2 ± 33.9 mg for Nanjing and Shanghai, respectively. One eighth of each membrane was used for solvent extraction of organic components. The remainder was dedicated to chemical analysis. For each city, samples from eight days were randomly pooled together for extraction. The membrane was cut into pieces, and then soxhlet extracted for 24 h in dichloromethane. The extract was dried under nitrogen sweeping. Then, the extracts were re-suspended at a stock concentration of 50 mg/mL in dimethyl sulfoxide (DMSO) to facilitate dissolution in cell media.

### Cell culture and treatment

A human bronchial epithelial cell line (BEAS-2B) was obtained from the China Center for Type Culture Collection (CCTCC). The cells were cultured in DMEM (Dulbecco’s modified Eagle’s medium) (HyClone, Logan, UT) supplemented with 10% FBS in a 5% CO_2_ humidified chamber at 37 °C. The human lung adenocarcinoma A549 cells (the Cell Bank of Type Culture Collection of Chinese Academy of Sciences) were maintained in DME/F12 (1:1) medium (HyClone, Logan, UT) supplemented with 10% FBS (fetal bovine serum) under the same condition. Both cells were exposed to organic extract of PM2.5 from both cities at the designated doses. The control group received the same extract from a null membrane. The dose of organic extract (5 μg/mL) equaled 10–20 μg/m^3^ of total PM2.5. These PM2.5 concentrations are in the regimen of the World Health Organization (WHO) annual standard^39^. In addition, this dose did not induce significant cellular toxicity as measured by MTT assay (Supplementary Figure 1). 3′,4′-dimethoxyflavone (DMF, AHR antagonist) and ellipticine (CYP1A1 inhibitor) were both co-incubated with PM2.5. Their final concentrations were both 10 μM.

### MTT assay

Cell viability was determined by MTT (Sigma-Aldrich, St Louis, MO, USA) colorimetric assay. The cells were seeded in 96-well plates at a density of 6,000 per well. The serial exposure concentrations were 0, 5, 10, 25, 50, 75 and 100 μg/mL. After an exposure period of 48 h, MTT was added, and the optical density (OD) was measured at 550 nm by a SpectraMAX M5 microplate reader (Molecular Devices, CA, USA). Cell viability was calculated as the relative value of the exposed group *vs* the control group.

### RNA isolation

Total RNA was obtained from cells by an RNA extraction kit (Omega Bio-Tek, Norcross, USA), according to the manufacturer’s instructions. RNA quality and quantity were determined on a nanophotometer (IMPLEN, GmbH, Germany). Then, equal amounts from each of the four replicates were pooled together for RNA sequencing.

### RNA sequencing and bioinformatics analysis

The noncoding RNA and mRNA were enriched from total RNA of BEAS-2B cells by removing rRNA. Then, they were fragmented into short fragments (approximately 200–500 nt). The fragments were used to synthesize first-strand cDNA by a random hexamer primer. PCR amplification was then performed to construct a library. The library was sequenced using the Illumina HiSeqTM 2000 at the Beijing Genomics Institute. Raw sequencing data from the control, Nanjing and Shanghai PM2.5-exposed groups were assembled and predicted independently. Then, all of the data were merged together to remove the redundant transcripts and to optimize the structure of the transcripts. Transcript assemblies were generated using *Cufflinks* ^40^. *Cuffcompare* was utilized to distinguish known and novel lncRNA transcripts, based on UCSC hg19 and NONCODE V3.0^41^. RNA expression levels were compared between different groups by *Cuffdiff*. RNAplex was adopted to predict antisense lncRNA-mRNA duplexes^42^. Pre-miRNA was predicted by miRBase^43^. The lncRNA family was predicted by the Rfam database^44^.

### Real-time RT-PCR

First strand cDNA was synthesized from total RNA using the SYBR Premix Ex Taq^TM^ kit (Takara, Dalian, China). The second PCR reaction was performed as follows: the thermal cycle of an initial denaturation step at 95 °C for 30 s, then 40 cycles at 95 °C for 5 s and 60 °C for 20 s on a Roche 480 instrument. A dissociation step was adopted to confirm the correct amplification of the target gene. Three replicates of each PCR reaction were performed for each tested gene. Glyceraldehyde-3-phosphate dehydrogenase (GAPDH) was used to normalize the expression levels of target genes. The relative expression levels of the tested genes were analyzed using the 2^−ΔΔCt^ method^45^. The primer sequences are listed in supplementary information 1 Table S1.

### Enzyme-linked immunosorbent assay (ELISA)

The culture medium was collected for ELISA after treatment of cells with PM2.5. Concentrations of both interleukin-6 (IL-6) and IL-8 were measured by ELISA following the manufacturer’s instructions (R&D systems, Minneapolis, MN).

### Cell invasion

Transwell assay was used to examine the cell invasion using a Matrigel invasion chamber with polycarbonate membranes (8-μm pores, Corning) coated with Matrigel (BD Biosciences). The invaded cells were fixed, stained, and counted by averaging ten fields under an Olympus CKX41 inverted microscope (Olympus, Tokyo, Japan).

### Determination of total PAHs and benzo(a)pyrene (BaP) toxic equivalents

The concentrations of sixteen priority PAHs were determined in PM2.5 by GC-MS/MS following our previous study^46^. The total BaP equivalent concentration was obtained by totaling the equivalence values of individual PAHs. The individual equivalence was calculated by multiplying the concentration by its BaP coefficient, according to the previous study^47^.

## Electronic supplementary material

supplementary information 1

supplementary information 2
