# Supplementary material for: Fine particulate matter 2.5 exerted its toxicological effect by regulating a new layer, long non-coding RNA
Source: Sci Rep. 2017 Aug 24;7:9392. doi: 10.1038/s41598-017-09818-6 (PMC5570922; doi:10.1038/s41598-017-09818-6)
Supplement: Supplementary file 1 — supplementary information 1 [file 41598_2017_9818_MOESM1_ESM.pdf]

Supplementary information 1

Fine particulate matter 2.5 exerted its toxicological effect by regulating a new layer, long non-coding RNA

Qiansheng Huang[a,b], Yulang Chi[a,b], Junjun Deng[a,b],Yiyao Liu[a,b], Yanyang Lu[a,b], Jinsheng Chen[a,b]*, Sijun Dong[a,b]*

a Center for Excellence in Regional Atmospheric Environment, Institute of Urban Environment, Chinese Academy of Sciences, Xiamen 361021, China
b Key Laboratory of Urban Environment and Health, Institute of Urban Environment, Chinese Academy of Sciences, Xiamen 361021, China

* Corresponding author. Tel/fax: +86 -592-6190779.

E-mail address: sjdong@iue.ac.cn, jschen@iue.ac.cn

Table of contents

Supplementary Material Table S1: Primer sequences used in the realtime RT-PCR…………………………………..…..…..…..…..…..……..………………3
Supplementary Material Table S2: Significantly enriched pathways from differentially expressed mRNA………………………..…..…..…..…..…..……..……….…………4
Supplementary Material Figure S1: Effects of organic extracts from Nanjing and Shanghai PM2.5 on the cell viability by MTT assay………………………………...5
Supplementary Material Figure S2: GO analysis of significantly differentially expressed mRNA between control *vs* Nanjing; control *vs* Shanghai…….….………...6

Table S1 primer sequences used in the realtime RT-PCR

Genes	Nucleotide accession number	Primer sequences used for RT-PCR (5' to 3')
n345347	/	F: GATGTTACAGCCTCTGCCTCTGG<br>R: GGATCCCAAGGAGGGAGAAAGGA
n405968	*NR_001458*	F: GCTCCTGGCACTGCAGATAACTT<br>R: GTGCCACCATTAGAGCCATCTCC
n407133	*NR_026545*	F: GCCTGAGCTTGCTCACCTTCAAA<br>R: CCTTGCCTCCTCCACATACTTCG
MALAT1<br>(metastasis associated lung adenocarcinoma transcript 1)	*NR_002819*	F: TAGGAAGACAGCAGCAGACAGG<br>R: TTGCTCGCTTGCTCCTCAGT
AFAP1<br>(actin filament associated protein 1)	*NM_001134647*	F: CCGTGCATCAACGGCTCGCTC<br>R: TTCACAACAGCCGCGGGATCC
AFAP1-AS1<br>(AFAP1 antisense RNA 1)	*NR_026892*	F: TCGCTCAATGGAGTGACGGCA<br>R: CGGCTGAGACCGCTGAGAACTT
IL-1β<br>(interleukin 1 beta)	*NM_000576*	F: GGACAAGCTGAGGAAGATGC<br>R: TCGTTATCCCATGTGTCGAA
IL-6<br>(interleukin 6)	*NM_000600*	F: AAATTCGGTACATCCTCGACGG<br>R: GGAAGGTTCAGGTTGTTTTCTGC
IL-8<br>(interleukin 8)	*NM_000584*	F: GCATAAAGACATACTCCAAACC<br>R: ACTTCTCCACAACCCTCTG
E-cadherin	*NM_004360*	F: GGATGTGCTGGATGTGAATG<br>R: CTGGGCAGTGTAGGATGTGA
N-cadherin	*NM_001308176*	F: CGTGAAGGTTTGCCAGTGT<br>R: GCACAAGGATAAGCAGGATG
HOXB-6<br>(homeobox B6)	*NM_018952.4*	F: TATGGTTCCAGAACCGACGCAT<br>R: TCGGCCTGTTTTTCTTCCTCCT
GAPDH<br>(Glyceraldehyde-3-phosphate dehydrogenase)	*NM_002046*	F: GGAGAAGGCTGGGGCTCAT<br>R: TGATGGCATGGACTGTGGTC

Table S2 significantly enriched pathways from differentially expressed mRNA (control *vs* Nanjing or control *vs* Shanghai) p<0.05

	C *vs* NJ		C *vs* SH	
	Name of pathway	Pathway ID	Name of pathway	Pathway ID
1	Amoebiasis	ko05146	Amoebiasis	ko05146
2	Rheumatoid arthritis	ko05323	Chemokine signaling pathway	ko04062
3	Rap1 signaling pathway	ko04015	Type II diabetes mellitus	ko04930
4	Chagas disease (American trypanosomiasis)	ko05142	Non-alcoholic fatty liver disease (NAFLD)	ko04932
5	Toll-like receptor signaling pathway	ko04620	Legionellosis	ko05134
6	Non-alcoholic fatty liver disease (NAFLD)	ko04932	Rheumatoid arthritis	ko05323
7	Melanoma	ko05218	Ovarian steroidogenesis	ko04913
8	Legionellosis	ko05134	Jak-STAT signaling pathway	ko04630
9	TNF signaling pathway	ko04668	Apoptosis	ko04210
10	PI3K-Akt signaling pathway	ko04151	Pertussis	ko05133

Figure S1

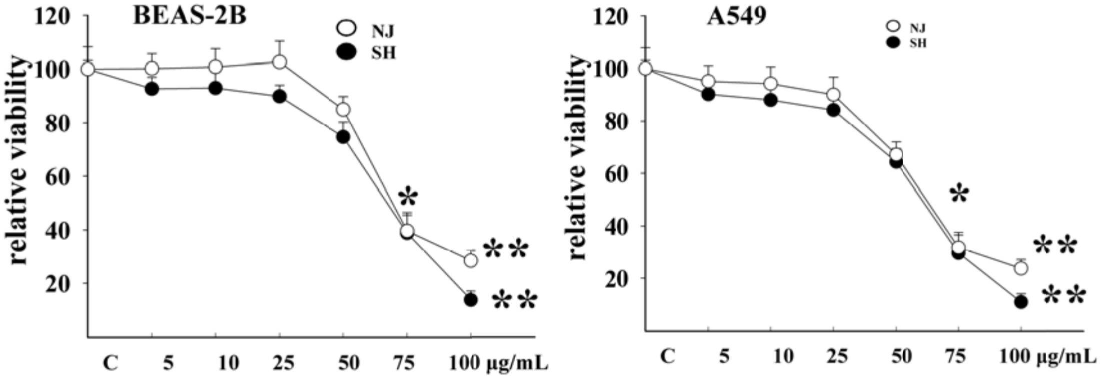

Figure S1 Effects of organic extracts from Nanjing and Shanghai PM2.5 on the viability of BEAS-2B and A549 cells by MTT assay. (*p<0.05, **p<0.01, ***p<0.001 *vs* control)

Figure S2

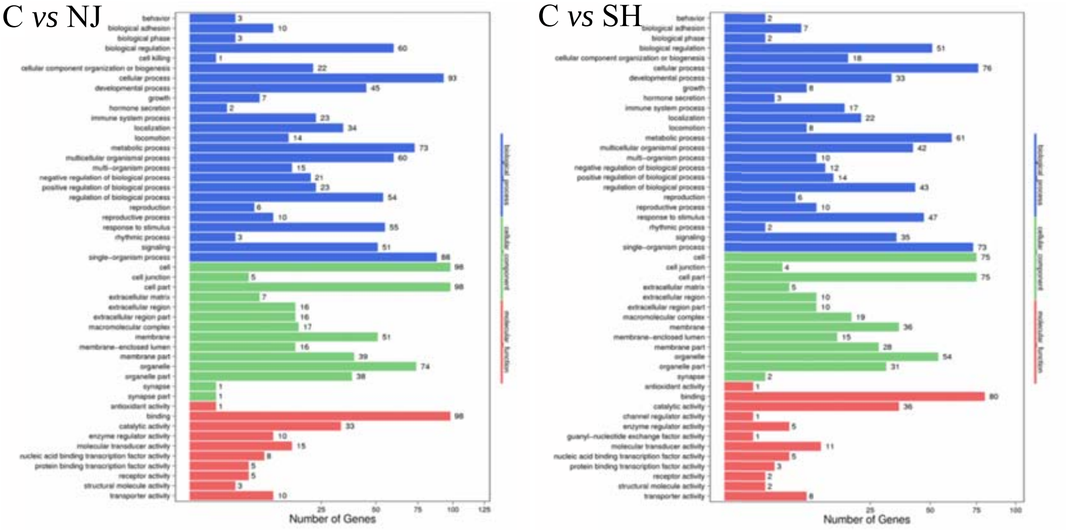

Figure S2 GO analysis of significantly differentially expressed mRNA between control *vs* Nanjing; control *vs* Shanghai.
